# A Digital Cephalometric Study on The Morphometric Evaluation of Soft Palate in Oral Submucous Fibrosis

**DOI:** 10.31557/APJCP.2020.21.7.2169

**Published:** 2020-07

**Authors:** Manaswita Tripathy, Jayaprasad Anekar, Raj AC, Sandeepa NC, Deepika Nappalli, Priya Lokanath, Abdulrahman Nahi Alharbi, Fahad Mohammed A Alsobil, Darshan Devang Divakar, Aftab Ahmed Khan, Chitra Jhugroo, Sanjeev Balappa Khanagar, Sachin Naik

**Affiliations:** 1 *Dental Surgeon, Sub-Divisional District Head Quarter (SDH) Gunupur District, Rayangada, Odisha, India. *; 2 *Department of Oral Medicine & Radiology, KVG Dental College & Hospital, Sullia, Karnataka, India.*; 3 *Department of Oral Medicine & Radiology, Mahe Institute of Dental Sciences & Hospital, Chalakkara, Pallor, Mahe - 673 310, U.T of Puducherry, India. *; 4 *Department of Diagnostic Sciences - Oral Biology, King Khalid University, College of Dentistry, Abha, KSA. *; 5 *General Dentist, MOH, Qaseem, KSA. *; 6 *General Dentist, MOH, Riyadh, KSA. *; 7 *Dental Biomaterials Research Chair, Dental Health Department, College of Applied Medical Sciences, King Saud University, Riyadh 11433, KSA. *; 8 *Dental Public Health, Preventive Dental Science Department, College of Dentistry, King Saud Bin Abdulaziz University for Health Sciences, Riyadh, KSA. *; 9 *King Abdullah International Medical Research Center, Ministry of National Guard Health Affairs, Riyadh, KSA . *

**Keywords:** Precancerous lesion, prevention, diagnosis

## Abstract

**Objective:**

Oral submucous fibrosis (OSMF) is a chronic precancerous condition affecting the oral cavity, which is progressive and characterised by burning sensation and fibrotic change leading to restriction of mouth opening. This study evaluated the morphology of soft palate in different stages of OSMF patients using digital lateral cephalogram and compare it with healthy individuals.

**Methods:**

The study included 60 subjects, who were grouped as 30 OSMF and 30 healthy subjects from the same geographic population. Digital lateral cephalograms were taken with Planmeca Proline XC (Oy, Helsinki, Finland). Soft palate morphology was evaluated using Lateral Cephalogram, and the results were analysed statistically.

**Results:**

Leaf-shaped (Type 1) soft palate was commonly seen in the control group and stage I and II OSMF. Stage III OSMF patients presented with a butt-shaped (Type 3) soft palate. As the disease progressed, there was a conversion of Type 1 variety of soft palate to Type 3 variety. There was a gradual reduction in the length of the soft palate in the anteroposterior direction in OSMF patients compared to the control group.

**Conclusion:**

Early cephalometric diagnosis of soft palate changes may play a pivotal role in the overall management of OSMF.

## Introduction

Oral submucous fibrosis (OSMF) is an insidious, progressive, irreversible and precancerous condition of the oral cavity characterised by a severe burning sensation, fibrotic change and difficulty in mouth opening (Misra et al., 1998; Tilakaratne et al., 2006; Rao, 2010). The disease primarily affects most of the oral cavity as well as oropharynx (Misra et al., 1998). There is extensive involvement of the hard and soft palate, including the uvula, which usually contracts to give a bud-like an appearance (Glick, 2015). The fibrosis of the mucosa in and around the uvula leads to characteristic abnormalities leading to a forward-pointing or a vanishing uvula (Chaturvedi, 2009). Morphological variations of soft palate play a key role, particularly in cleft palate and obstructive sleep apnea (Nelson et al., 2008). There is a general belief that the oropharyngeal and nasopharyngeal structures play an essential role in the development and functioning of the dentofacial complex (Kaur et al., 2014).

Cephalogram is an essential diagnostic tool to assess the hard and soft tissues of the maxillofacial area and for the analysis of nasopharynx (Bitar et al., 2010). It is advantageous because it is economical, noninvasive, correlates with investigation such as computed tomography and can be achieved with less radiation (Samman et al., 2003). Cephalometric evaluation assists in the analysis of dental, skeletal anomalies and soft tissue structures and forms (Bhad et al., 2013). Morphometric assessment of the soft palate and the configuration of adjacent structures in-depth and height in the midsagittal plane can be defined in a Lateral Cephalogram (Santosh et al., 2015).

Cephalometric analysis is one of the most generally recognised techniques for evaluating the soft palate. In the past, diversity in uvular morphology was not considered as an area of significance. The present study is carried out to assess the soft palate morphology in OSMF patients using digital Lateral Cephalogram (Bejdová et al., 2012). Abramson et al., (2010) and Vizzotto et al., (2011) correlated the three-dimensional CT findings of the size and shape of the airway with lateral cephalometric dimensions. It was observed that three-dimensional CT and lateral cephalometric measurements were reproducible and reliable (Abramson et al., 2010; Vizzotto et al., 2012).

## Materials and Methods

*Sample*

The total study group consisted of 60 patients who were divided into two groups, Group 1 (control group) consisted of 30 healthy patients, Group 2 (study group) consisted of 30 patients with different stages of OSMF (stage I, stage II, stage III) 10 patients of each stage. The clinical diagnosis of OSMF was confirmed by history and clinical examination. The OSMF was staged according to Nagesh and Durgesh bailoor classification (More et al., 2012). According to this classification, OSMF patients were categorised into stage I, stage II, and stage III. Stage or early OSMF present with mild blanching, no limitation in mouth opening with an average distance between maxillary and mandibular central incisor tips is considered to be 35 to 45 mm in males and 30 to 42 mm in females. No restriction in tongue protrusion is also a feature included under stage I. Stage II or moderate OSMF present with moderate to severe blanching, reduced mouth opening by 33%, demonstrably diminished flexibility of cheek, palpable bands, burning sensation without any stimuli, either unilateral or bilateral lymphadenopathy, and anemia on haematological examination. Stage III patients or severe OSMF were staged based on the features of very severe burning sensation, unable to perform daily work, more than 66% decrease in the mouth opening, flexibility of cheek, and tongue protrusion. The tongue may appear to be fixed. Ulcerative lesions on the mucosa, thick, palpable bands and bilateral lymphadenopathy may also manifest (More et al., 2012). 

Patients with a known history of surgery in the area of the palate, cleft lip, cleft palate and scleroderma were not included in the study. Informed consent was obtained from the patients before the documentation and radiographic procedures. Each subject from both groups was subjected to Digital Lateral Cephalometric imaging using PlanmecaProline XC (Oy, Helsinki, Finland). Standard exposure parameters were followed while taking the cephalogram, i.e., at 68KvP, 15mA and 9.4 seconds exposure time. The subjects were positioned in Cephalostat with Frankfort plane parallel to the floor. They were instructed swallowing saliva to clear the oral cavity and pharynx. Patients were asked to close their mouth lightly and to place their upper and lower teeth in centric occlusion with their oropharyngeal musculature relaxed. 

*Cephalometric evaluation*

The following parameters on the morphology of the soft palate were studied.

**Figure 1:**
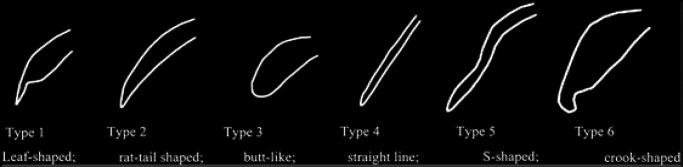
Morphology of the Soft Palates According to You et al

• Morphology of soft palates was observed and classified based on the classification given by You et al. (You et al., 2008). It was classified as followed: Type 1 (leaf-shaped), Type 2 (rat-tail shaped), Type 3 (butt-like), Type 4 (straight line), Type 5 (S-shaped) and Type 6 (crook-shaped) (Figure 1).

• The length of the soft palate was evaluated by measuring the linear distance from the Posterior Nasal Spine (PNS) to the tip of the uvula of the resting soft palate. 

• Supero inferior dimension or the width of the soft palate was measured at the thickest area of the soft palate. 

*Statistical analysis*

The statistical software IBM SPSS version 24 (IBMSPSS, Chicago, IL, USA) and Microsoft Excel^®^ was used to perform all statistical analyses. Statistically, significance was considered at p<0.05 level. The results were tabulated and analysed statistically using the chi-square test, and cross-tabulation was done to evaluate the shape of the soft palate in various study groups using the student t-test. 

## Results

**Figure 2:**
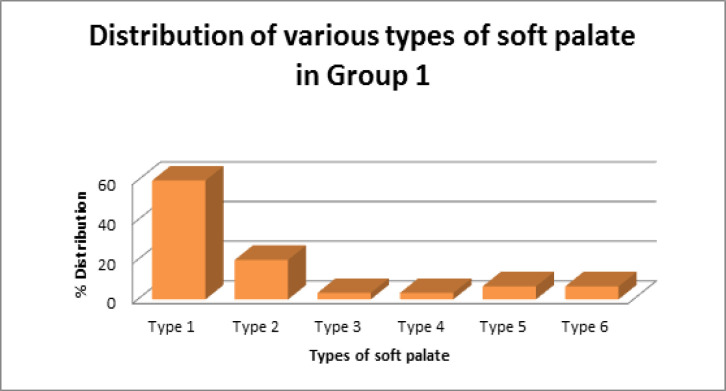
Percentage Distribution of Types of Soft Palate in Group 1

**Figure 3.**
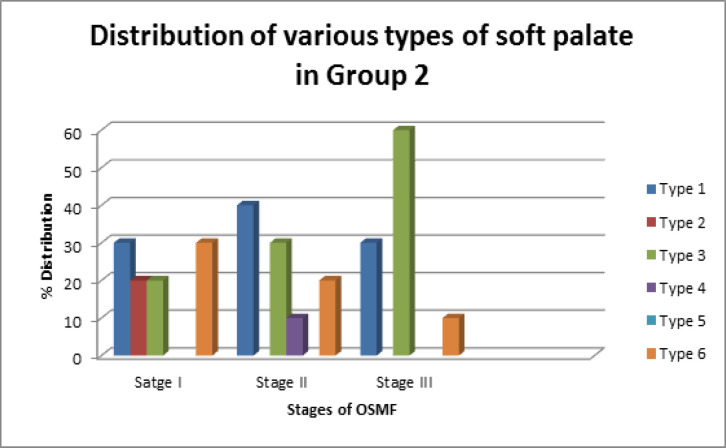
Percentage Distribution of Types of Soft Palate in Group 2

Group 1 (control group) constituted 30 healthy subjects, and Group 2 included OSMF patients. Out of 60 subjects, 61.6% (37) were male, and 38.3% (23) were females. Mean age in Group 1 (control group) was 33.8 years and Group 2 (OSMF patients) was 35.5 years. Among Group 1, 60% (18) of individuals had leaf-shaped soft palate (Type 1). Type 2 soft palate was seen in 20% (6) of healthy subjects. Type 3 and Type 4 was seen in 3.3% (1) of the individual. Type 5 and Type 6 soft palate was observed in 6.6% (2) of Group 1. Type 1 was the most common soft palate type in Group 1 (control group) followed by Type 2, Type 5, Type 6, Type 3 and Type 4. Among OSMF patients, soft palate morphology was observed, stage I OSMF patients presented with the following pattern: Type 1in 30% (3), Type 2 and Type 3 in 20% (2) and Type 6 in 30% (3). Type 4 and Type 5 was not observed in stage 1 patients. Among stage II OSMF patients, 40% (4) presented with Type 1, 30% (3) with Type 3 and 10% (1) with Type 4. Type 6 soft palate was seen in 20% (2). Type 2 and Type 5 were not observed in stage II patients. Among stage III OSMF patients, 30% (3) had Type 1, 60% (6) had Type 3 and 10% (1) with Type 6 soft palate (Figures 2 and 3).

Cross-tabulation was done to evaluate the shape of the soft palate in various study groups using the student t-test. Leaf shape Type 1 was the most common form of the soft palate in Group 1, and the least common was Type 3 and Type 4. In Group 2, Type 1 was the most common form observed in stage I and stage II, while Type 3 was more common in stage III. It was noticed that as the disease progressed from stage I to stage III, butt like the shape of soft palate became more frequent, which was statistically significant (p<0.05). It shows that with advancing clinical stage of OSMF, the incidence of Type 3 soft palate increases.

**Figure 4:**
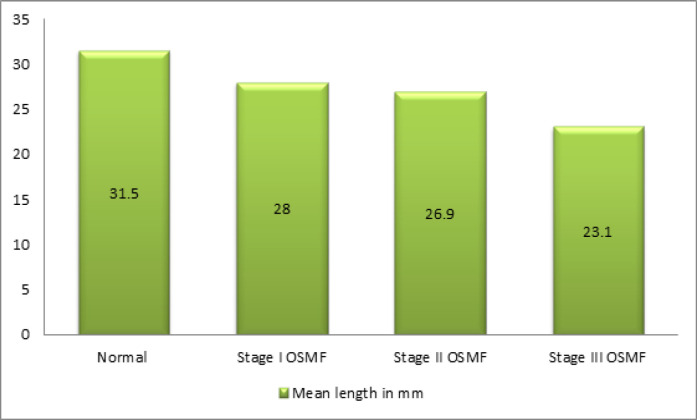
Comparison of Mean Length of the Soft Palate in Healthy Individuals and Different Stages of OSMF

**Figure 5:**
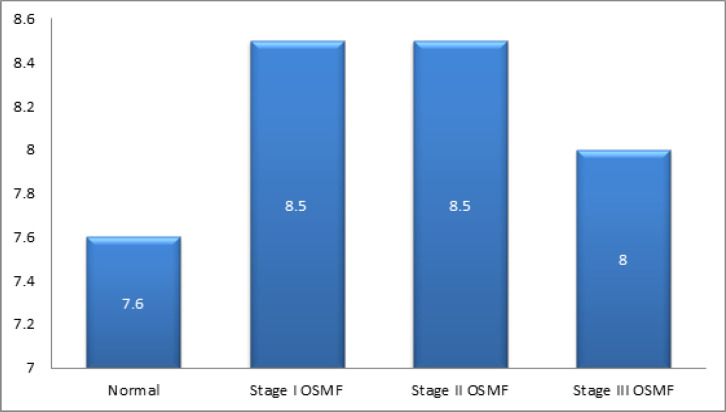
Comparison of Mean Width of the Soft Palate in Healthy Individuals and Different Stages of OSMF

Length of the soft palate was measured from the radiograph. The mean length of the soft palate in Group 1 (control patients) was 31.5 mm. Mean length was 28.0mm in stage I OSMF patients. In the case of stage II OSMF patients, length of the soft palate was found to be 26.9mm, and in stage III OSMF patients, it was 23.1mm. It was observed that the length of the soft palate was gradually decreased as the disease progressed (Figure 4). The decrease in the length of soft palate according to the stage of OSMF was found to be statistically significant (p<0.05). Mean width of the soft palate in Group 1 (control group) was 7.6mm ± 1.3. In stage I OSMF, the width was found to be 8.5mm ± 1.1. It was 8.5mm ± 0.8 in stage II OSMF and 8.0mm ± 0.6 in stage III OSMF subjects (Figure 5). There was a statistically significant increase in the width of the soft palate in stage I and stage II OSMF patients compared to healthy individuals. Mean length of the soft palate in Group 1 and Group 2 patients according to the type of soft palate was tabulated. For Type 1 soft palate, mean length in Group 1 (control group) was 31.6mm, while in Group 2, it was 26.9mm, and the difference was statistically significant. Similar findings were noticed for Type 2 and Type 6 soft palates where the difference in the mean length between the groups was statistically significant (Table 1). Similarly mean superoinferior dimensions of the soft palate in Group 1 and Group 2, according to the type of soft palate was tabulated. For Type 1 soft palate, mean width in Group 1 was 7.9 mm, while in Group 2 it was 8.8mm and the difference was statistically significant. Similar findings were noticed for Type 3 variety of soft palates (Table 2). Mean length of the soft palate in anteroposterior direction among different stages of OSMF patients according to the type of soft palate was also compared. For Type 1 soft palate, mean length in stage, I OSMF patients was 29.4mm. Among stage II patients, it was 26.7mm, and stage III OSMF had a length of 24.9mm, and the difference was statistically significant. Similar findings were noticed for Type 3 soft palate where the difference in the mean length in anteroposterior direction was statistically significant among OSMF staging (Table 3). Width of the soft palate in different soft palate type was compared among different stages of OSMF. For Type 1 soft palate, mean length in a superoinferior direction in stage I was 9.1mm, stage II was 8.7mm, stage III was 8.6mm, and the difference was statistically significant. For Type 3 soft palate, the width of the soft palate in stage 1 OSMF was 8.9mm, 9.2mm in stage II and 7.8mm in stage III OSMF. For Type 6 soft palate, the width of the soft palate in stage I was 8.8mm, 7.7mm in stage II and 7.5mm in stage III. The difference in the mean length in the superoinferior direction was statistically significant in Type 3 and Type 6 soft palate (Table 4).

**Table 1:** Comparison of the Mean Length of the Soft Palate in Group 1 (Control) and Group 2 (OSMF) According to the Types of the Soft Palate

Type of soft palate	Study	Mean length in A-P (mm)	SD	*P*-value
Type 1	Group 1	31.6	3.8	0
	Group 2	26.9	2.6	0
Type 2	Group 1	30.9	4.4	0
	Group 2	27.7	0.7	0.011
Type 3	Group 1	32.9	-	-
	Group 2	24.6	4.2	
Type 4	Group 1	26.6	-	-
	Group 2	26.6	-	
Type 5	Group 1	30.25	-	-
	Group 2	-	-	
Type 6	Group 1	30.2	2.6	0.04
	Group 2	26.4	1.1	0

**Table 2:** Comparison of the Mean Width of the Soft Palate in Group 1 and Group 2 According to the Types of the Soft Palate

Type of soft palate	Study	Mean length in S-I	SD	*P*-value
Type 1	Group 1	7.9	1.3	0
	Group 2	8.8	0.6	
Type 2	Group 1	6.5	0.9	0
	Group 2	6.7	0.6	0.042
Type 3	Group 1	9.5	-	0
	Group 2	8.4	0.7	
Type 4	Group 1	6.8	-	-
	Group 2	7.6	-	
Type 5	Group 1	8.2	0.1	-
	Group 2	-	-	
Type 6	Group 1	7.9	2.6	0.15
	Group 2	8.2	0.9	0

**Table 3:** Comparison of Mean Length of the Soft Palate among Different Stages of OSMF

Type of soft palate	Study	Mean length in A-P	SD	*P*-value
Type 1	Stage I OSMF	29.4	1.4	0.001
	Stage II OSMF	26.7	1.1	0
	Stage III OSMF	24.9	3.4	0.006
Type 2	Stage I OSMF	27.7	-	-
	Stage II OSMF	-		
	Stage III OSMF	-		
Type 3	Stage I OSMF	28.8	0.5	0.009
	Stage II OSMF	27.4	1.5	0.001
	Stage III OSMF	21.9	3.8	0
Type 4	Stage I OSMF	-	-	-
	Stage II OSMF	26.6	-	
	Stage III OSMF	-	-	
Type 5	Stage I OSMF	-	-	-
	Stage II OSMF	-	-	
	Stage III OSMF	-	-	
Type 6	Stage I OSMF	26.4	0.8	0
	Stage II OSMF	27.1	1.5	0.026
	Stage III OSMF	32.2	-	

**Table 4:** Comparison of Mean Width of the Soft Palate among Different Stages of OSMF

Type of soft palate	Study	Mean width in S-I	SD	*P*-value
Type 1	Stage I OSMF	9.1	0.5	0.001
	Stage II OSMF	8.7	0.6	0
	Stage IIIOSMF	8.6	0.6	0.002
Type 2	Stage I OSMF	6.7	-	-
	Stage II OSMF	-	-	
	Stage III OSMF	-	-	
Type 3	Stage I OSMF	8.9	0.3	0.018
	Stage II OSMF	9.2	0.3	0
	Stage III OSMF	7.8	0.4	0
Type 4	Stage I OSMF	-	-	-
	Stage II OSMF	7.6	-	
	Stage III OSMF	-	-	
Type 5	Stage I OSMF	-	-	-
	Stage II OSMF	-	-	
	Stage III OSMF	-	-	
Type 6	Stage I OSMF	8.8	0.9	0.004
	Stage II OSMF	7.7	1	0.061
	Stage III OSMF	7.5	-	-

## Discussion

Oral submucous fibrosis (OSMF) is an insidious, chronic disease affecting any part of the oral cavity (Kumar and Gopal, 2011; Mohan et al., 2014), and sometimes the pharynx. OSMF has been previously termed as idiopathic scleroderma of mouth, idiopathic palatal fibrosis, juxta-epithelial fibrosis and sclerosing stomatitis (Rajendran, 1994). The hallmark of the disease is submucosal fibrosis that affects most parts of the oral cavity, progressive trismus due to stiff lips, cheeks, pharynx and upper third of the esophagus leading to dysphagia (Gupta et al., 2008; Darshan Devang Divakar, 2013). Oral submucous fibrosis is one such pathology that could alter the dimensions of soft tissue components like oral mucosa and soft palate; in this aspect, the present study aimed to evaluate the morphological patterns of the soft palate in both healthy and oral submucous fibrosis individuals. The soft palate plays a very critical part in an approximation of soft palate with pharyngeal walls. This sphincteric mechanism separates the nasal cavity and the oral cavity during functions such as speech and deglutition (Johns et al., 2003). In this context, the study of these soft palate patterns like shape, length, and width help for the evaluation of any risk factors for velopharyngeal incompetence.

In the present study, the classification used to stage the OSMF patients was based on the classification proposed by Nagesh and Bailoor. OSMF patients were classified into stage I, stage II and stage III. Ten subjects were included in the study from each stage of the disease. Thirty healthy subjects who require lateral cephalogram for the diagnostic purpose was included in the control group (Group 1)

Cephalometry is comparatively an inexpensive, noninvasive radiographic method which allows a proper assessment of the soft tissue components of the soft palate and its surrounding structures. The limiting factor for using the lateral cephalometric radiography is the fact that it offers only two-dimensional images of the airway (Martin et al., 2006). Computed tomography (CT), which provides a three-dimensional image, could give a detailed analysis of the relationship between the upper airway and its surrounding soft tissues (Lowe et al., 1995). Previous studies of comparison of CT and Lateral Cephalogram in providing a comparable result have suggested the use of Lateral Cephalogram in a routine set up. Previous studies have assessed the dimensional changes of the soft palate with increasing age, cleft lip and palate and in patients with sleep apnea using cephalometry, whereas studies regarding OSMF were inadequate (Tanwir and Akhlaq, 2011). Radiological investigations were much helpful for the diagnosis of various pathologies of the soft palate like neoplastic, neurologic, and inflammatory conditions. Morphological variants of soft palate play a very critical role, particularly in conditions like cleft palate and obstructive sleep apnea (Shimomatsu et al., 2012).

*Type of soft palate among OSMF patients and control group*

In our study, Group 1 (control group) presented most commonly with Type 1 soft palate, which was seen in 60% of individuals. It was followed by Type 2, Type 5, Type 6, Type 3 and Type 4 soft palate in decreasing frequency. It was in accordance with other studies of You et al., (2008); Kumar and Gopal,(2011); Verma et al., (2014) and Khare et al., (2019) where leaf-shaped soft palate was described as the most common variant. These studies also showed that crook shaped type is the least typical soft palate which was not a finding in our study. Type 2 was the most common soft plate type in a study by Raja Lakshmi et al., (2016) and Praveen et al. According to More et al. butt type of morphology were most common in healthy individuals (More et al., 2015).

In OSMF patients, when staging was not considered, common soft palate type observed was Type 1 followed by Type 3 and Type 6. This was in accordance with the study by Domir et al., (2019) where Type 1 was the most common soft palate type. It was followed by Type 2 and Type 6. Type 1 was also observed as the most common type in studies of Chintamaneni et al., (2016); Shankar et al., (2014); Mohan et al., (2014) and Tekchandani et al. , (2015). Agrawal et al., (2016). in his study found that rat-tail shape to be more common in OSMF patients (Agrawal, 2016).

In our study, Type 4 was the least common type seen in OSMF patients, and Type 5 was not observed in Group 2 patients. This was in accordance with Shankar et al. study (Shankar et al., 2014). Type 4 was observed in only one case among stage II OSMF. In our study, Type 1 and 6 was the most common soft palate found in stage I OSMF, followed by Type 2. Stage 2 OSMF patients presented commonly with Type 1 soft palate, followed by Type 3 and Type 6. Stage 3 OSMF patients had Type 3 soft palate commonly followed by Type 1 and Type 6. As in our study, Type 3 was reported as a common type in later stages of OSMF by Rathore S et al (Rathore S, 2019).

In another study, where the same classification was used for staging the patients. Stage 1 OSMF patients presented with most commonly Type 4 followed by Type 2 and Type 1. Stage 2 OSMF patients showed most of Type 1 followed by Type 2, Type 4 and Type 3. Stage 3 patients had Type 6 the most common, followed by Type 3 and Type 1. Type 4 was commonly observed among OSMF patients in contrast to our study (Raja Lakshmi et al., 2016).

*Length of soft palate among OSMF patients and control group*

There is a significant decrease in the length of the soft palate in anteroposterior direction in the study group when compared with the control group. It was consistent with findings by Rathore et al., (2019); Chintamaneni et al., (2009). There was a reduction in the length as it goes from stage I to stage II. This was consistent with other studies. In each type of soft palate, mean length of the soft palate in control was higher than OSMF patients except in case of Type 4 soft palate were in length was the same in both control and OSMF group. It was also observed in our study that there was a gradual shortening of the soft palate with the progression of OSMF from stage I to stage III especially in case of Type 1 and Type 3 which was statistically significant. It was in accordance with the study by Shankar et al., (2014) and Chintamaneni et al., (2009). Among Type 6 soft palate in our study, there was no mentioned correlation was observed in length as the disease progressed from stage I to stage III, which was in contrast to Chintamaneni (Raja Lakshmi et al., 2016).

*Width of the soft palate in OSMF and control group*

The results of the present study showed that the width of soft palate increased in OSMF individuals as compared to healthy individuals except in case of Type 3 soft palate where the width was reduced in OSMF patients compared to control Group. There was a decrease in width in individuals with OSMF from stage I to stage III with a statistically significant difference. A few other studies demonstrated a minor increase in width as the disease progress, and our study presented a different result. The changes in length in superoinferior direction was not marked as compared to the anteroposterior direction in Shankar et al. study (Shankar et al., 2014; Raja Lakshmi et al., 2016).

In our study, there was a gradual change in the dimensions and patterns of the soft palate with the advance in the staging of OSMF, and interestingly a negative correlation was observed in the staging of OSMF and anteroposterior and superoinferior dimension. As some of the soft palate types were not seen in both groups, validation of result need a larger sample size to establish the suggested association. 

In conclusion, early cephalometric diagnosis of soft palate changes may play a pivotal role in the overall management of OSMF. Further study with larger sample size and longer duration of follow up with post-treatment changes in soft palate is required to know more about this disease.

## Availability of material

The datasets analysed during the present study are online available in the public database.
